# Clinical safety and efficacy of a preloaded monofocal hydrophobic acrylic intraocular lens in a real-world population

**DOI:** 10.1186/s12886-021-02142-8

**Published:** 2021-10-25

**Authors:** Samuel Giles Latham, Francis Carr, Hala Ali, Vinod Gangwani

**Affiliations:** Ashford and St Peter’s Hospitals NHS Foundation Trust, Ashford, UK

**Keywords:** Cataract, Intraocular lens (IOL), Preloaded, Hydrophobic

## Abstract

**Background:**

This study was designed to evaluate visual, refractive and safety outcomes in eyes after they underwent phacoemulsification and implantation of a preloaded monofocal hydrophobic acrylic intraocular lens.

**Methods:**

This was a single center observational study conducted at Ashford and St Peter’s Hospitals NHS Foundation Trust, United Kingdom. Patients were included if they had cataract extraction with in-the-bag implantation of the EyeCee® One preloaded intraocular lens from August to October 2019. Pre-operative, surgery-related and 2 weeks and 3 months post-operative data was collected. Surgeons at this trust were then asked to complete a feedback form to evaluate their experience of implanting the EyeCee® One.

**Results:**

One hundred fifty-two eyes were included in the study. Ninety-four (62%) of these eyes had cataract but no concomitant ocular pathology that could potentially affect visual acuity. Three months post-operatively, 98.7% of all eyes had monocular CDVA ≤0.3 logMAR. 100% of the eyes without concomitant ocular pathology achieved this target. The mean CDVA of all eyes in this study improved from 0.43 ± 0.43 logMAR pre-operatively, to 0.05 ± 0.11 logMAR post-operatively (*p* < 0.05). The mean sphere and spherical equivalent values showed significant improvements (*p* < 0.05) and (*p* < 0.05). There were no intraoperative complications and 1.3% of patients reported complications 2 weeks post-operatively. All of the participating surgeons said they would use the EyeCee® One again with 64% providing an overall rating of ‘excellent’ for their experience of implanting this intraocular lens.

**Conclusions:**

This study indicates excellent post-operative visual acuity and refractive outcomes in eyes after EyeCee® One implantation. This is accompanied with very little risk of intraoperative and post-operative complications.

## Background

Intraocular lenses (IOLs) are a key component of modern-day cataract surgery and are used as an artificial replacement for the natural crystalline lens. Today, there are various types of IOLs on the market and ophthalmologists are able to select the most appropriate IOL given the clinical circumstances. For accurate decision making, it is integral to acknowledge that each model of IOL provides its own qualities and limitations. A recent advancement in cataract surgery has been the introduction of preloaded IOL injector systems. In general, IOL implantation using injector systems requires a smaller incision size than IOL implantation using forceps. This results in quicker wound healing and reduced risk of infection [1, 2]. Preloaded IOL injector systems were designed to streamline, simplify and standardize the surgery preparation process. They avoid manual loading errors and damages, shorten operation time, reduce the number of surgical instruments required, lower cost, minimize complexity, and decrease risk of instrument contamination [3, 4]. The risk for bacterial entry into the eye is also reduced as the foldable IOL makes no direct contact with the surgical incision or the operative field.

The most common long-term complication of cataract surgery is posterior capsule opacification (PCO) [5]. Amongst other factors, the incidence of PCO is affected by the biomaterial used in IOL manufacturing. Currently, foldable IOLs for small-incision cataract surgery are manufactured using acrylic or silicone biomaterials. IOLs made from acrylic biomaterials exhibit lower incidence rates of PCO and better overall stability [6]. Acrylic biomaterials are typically categorized according to their hydrophobic or hydrophilic surfaces. Comparative studies have shown that acrylic IOLs with hydrophobic surfaces are even less likely to cause PCO and are associated with better capsular biocompatibility than their hydrophilic counterparts [7, 8]. This reduced likelihood of postoperative complications and subsequent correction procedures has led to hydrophobic acrylic IOLs becoming the most used today [9].

Bausch + Lomb (Quebec, Canada) manufactures the EyeCee® One, which is a preloaded monofocal hydrophobic acrylic IOL that has aspheric optic, 360-degree posterior square edge, and modified L-loop haptics design features. The material specifications include UV and blue-light filters, a dense polymer network that is produced by a double-polymerization manufacturing process, and a refractive index of 1.52. In this study we aim to assess the real-world outcomes and the intraoperative performance of the EyeCee® One as part of routine cataract surgery in an NHS Foundation Trust.

## Methods

This open-label, non-interventional, observational study was performed at a teaching hospital in the United Kingdom. Ashford and St Peter’s Hospitals NHS Foundation Trust Institutional Review Board approval was obtained following a review of the study protocol (registration number: TASCC Ophth (2020–08)). The study was exempt from UK National Research Ethics Service approval (as per NHS Health Research Authority guidance). Permission was obtained to access patient data, which was de-identified. Informed consent was obtained from all subjects.

### Patient population

Patients included in this study had cataract extraction with in-the-bag implantation of the EyeCee® One preloaded intraocular lens between 1st August 2019 and 31st October 2019 at Ashford and St Peter’s Hospitals NHS Foundation Trust, United Kingdom. The patients were divided into two groups depending on the presence or absence of a concomitant ocular pathology that could potentially affect visual acuity.

### Surgical technique

Surgery was performed by experienced surgeons with at least 5 years of practice. Choice of IOL calculation formula was left to each surgeon’s discretion, enabling better representation of real-world conditions. Self-sealing 2.4-mm corneal incision (as per the standard keratome used at the Trust), capsulorhexis and conventional phacoemulsification was used in all cases. The EyeCee® One was implanted in the capsular bag using the preloaded injector combined with ophthalmic viscosurgical device. Residual ophthalmic viscosurgical device was subsequently removed from the bag. The corneal incisions were sealed with stromal hydration and intracameral cefuroxime was administered. Post-operatively, all patients received topical dexamethasone 0.1% eyedrops 4 times a day for 6 weeks and topical chloramphenicol 0.5% eyedrops 4 times a day for 2 weeks. All patients were reviewed at 2 weeks for complications.

### Data collection

Pre-operative, surgery-related and 2 weeks and 3 months post-operative data was collected prospectively. Pre-operative data collection included ocular history, monocular uncorrected and corrected distance acuities, refraction (obtained either by manifest refraction by an optometrist or by autorefraction) and biometry (swept-source optical coherence tomography biometry using Zeiss IOLMaster 700). Surgery-related data included intraoperative complications, IOL power and expected post-operative refraction. Complications, including IOL decentration were collected at review 2 weeks after surgery. Monocular corrected distance acuity and refraction (obtained by manifest refraction by an optometrist) was collected 3 months after surgery with 6-m Snellen charts. Implanting surgeons were asked in August 2020 to complete a feedback form to evaluate their experience of implanting the EyeCee® One. This form was self-made and consisted of a 5-point scale (1 being poor, 5 being excellent) to evaluate ease of loading, ease of introduction, control of lens injection, ease of placement into capsular bag, centration, and overall rating.

### Statistical analysis

Statistical analysis was performed using SPSS Statistics Version 26 (IBM, USA). Quantitative endpoints are presented as mean ± standard deviation (SD) and range (minimum, maximum). Qualitative endpoints are presented as numbers and percentage of each modality and number of patients. For quantitative endpoints, Wilcoxon signed rank test for non-parametric tests was utilized. In all cases, a *p*-value less than 0.05 was considered statistically significant.

## Results

### Patient characteristics

One hundred fifty-two eyes (75 right, 77 left) of 105 patients (56 male, 49 female) with a mean age of 76.1 years (SD 7.8, range 49–92) were included in the study (see Table 1).

**Table 1:** Pre-operative characteristics

	Mean	SD	Range
Age (years)	76.1	7.8	49; 92
Eyes (% right/left)	49/51		
Gender (% men/women)	53/47		
Axial length (mm)	23.88	1.08	21.77; 26.39
IOL power	21.28	3.35	13; 28
Formula	Haigis 9 (5.9%)		
	Haigis-L 1 (0.7%)		
	Holladay2 10 (6.6%)		
	SRK/T 132 (86.8%)		
Sphere	−0.88	2.99	−10.50; + 7.75
Cylinder	−0.63	1.22	−3.75; + 3.50
Spherical equivalent	−1.19	3.00	−8.75; + 8.50
UDVA	0.63	0.51	0.00; 2.30
CDVA	0.43	0.43	0.00; 2.30

Amongst the eyes, two subpopulations were defined. The ‘healthy eye’ group included 94 eyes (62%) with cataract but without any ocular pathology that could potentially affect visual acuity. The ‘pathological eye’ group included 58 eyes (38%) with at least one concomitant ocular pathology potentially affecting visual acuity (see Table 2).

**Table 2:** Pathological eyes

	Number of eyes
Diabetic retinopathy	22 (37.9%)
Dry age-related macular degeneration	21 (36.2%)
Sicca syndrome	8 (13.8%)
Glaucoma	6 (10.3%)
Ectropion	3 (5.2%)
Epiretinal membrane	2 (3.4%)
Vitreomacular traction	2 (3.4%)
Macular hole	1 (1.7%)
Branch retinal vein occlusion	1 (1.7%)
Amblyopia	1 (1.7%)
Laser retinopexy	1 (1.7%)
Refractive surgery	1 (1.7%)
Allergic eye disease	1 (1.7%)

### Surgeons

Phacoemulsification and EyeCee® One implantation was performed by 21 different surgeons (17 consultants, 3 fellows, 1 specialty registrar).

### Visual acuity

**Fig. 1:**
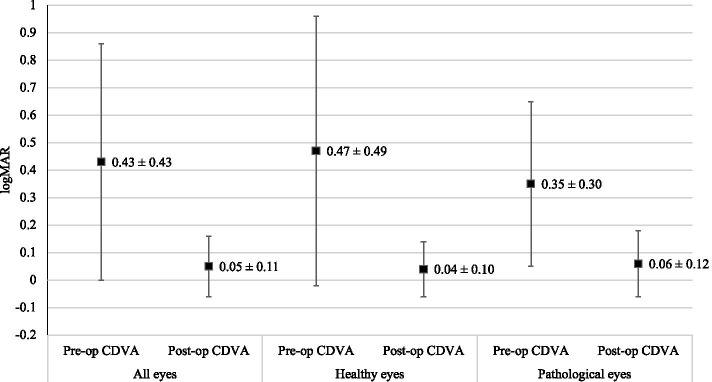
Changes in monocular CDVA (mean ± SD)

**Fig. 2:**
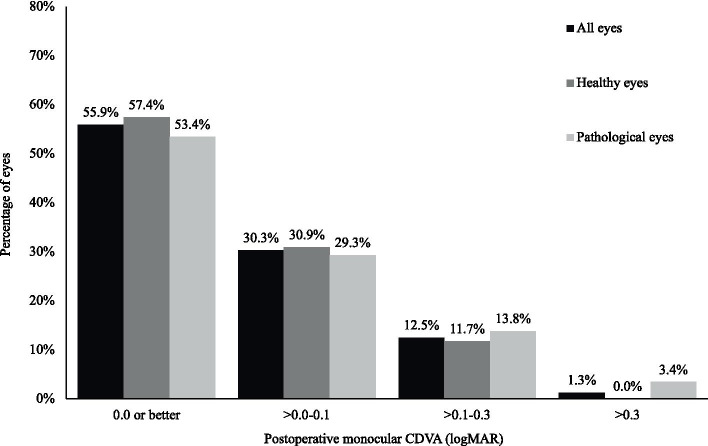
Post-operative monocular CDVA

Statistically significant improvements were seen in the mean CDVA of ‘healthy eyes’ and ‘pathological eyes’ 3 months post-operatively (*p* < 0.05) (see Fig. 1). The mean CDVA of all eyes improved from 0.43 ± 0.43 logarithm of the minimum angle of resolution (logMAR) pre-operatively, to 0.05 ± 0.11 logMAR 3 months post-operatively (*p* < 0.05). 98.7% of these postoperative eyes had monocular CDVA equal or better than 0.3 logMAR. 100% of the eyes in the ‘healthy eyes’ population achieved this target, whereas two eyes in the ‘pathological eyes’ group had monocular CDVA worse than 0.3 logMAR (see Fig. 2). Both of these eyes had dry age-related macular degeneration (AMD) with geographic atrophy. Of the entire cohort, 12 eyes (7.8%) had no change in visual acuity following cataract surgery and one eye (0.7%) experienced a loss in visual acuity. This loss was attributed to progression of dry AMD with geographic atrophy and amounted to 0.10 logMAR.

### Refraction

**Fig. 3:**
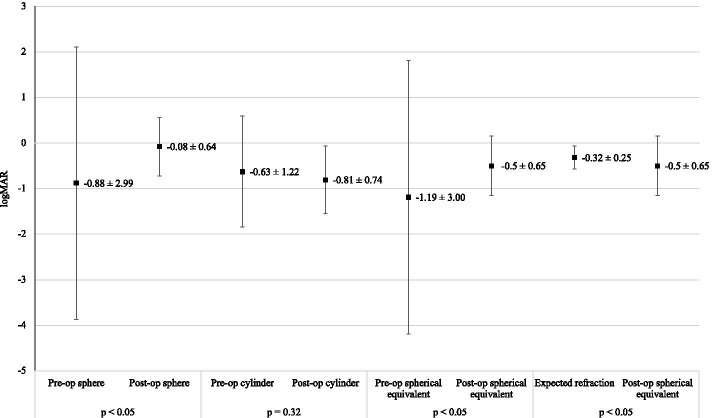
Changes in refraction (mean ± SD)

Pre-operatively, all eyes in the study had a mean sphere, mean cylinder and mean spherical equivalent of − 0.88, − 0.63 and − 1.19, respectively. Three months post-operatively, all eyes in the study had a mean sphere of − 0.08, a mean cylinder of − 0.81, and a mean spherical equivalent of − 0.50. The expected refraction of all eyes in this study had a mean value of − 0.32. There was a statistically significant difference between expected refraction and post-operative spherical equivalent (*p* < 0.05) (see Fig. 3).

### Safety

There were no cases of posterior capsule rupture, zonular dialysis, endophthalmitis or IOL crimping during or after IOL implantation. All 152 (100%) IOLs were centered at 2 weeks post-operative under the slit-lamp. Regarding complications noted at this point, one (0.7%) patient demonstrated allergic conjunctivitis and one (0.7%) patient had entropion. As stated above, one (0.7%) eye experienced visual acuity loss at 3 weeks post-operative, which was not attributed to cataract surgery.

### Ease of use

Fourteen of the twenty-one operating surgeons completed a feedback form that was designed to evaluate the EyeCee® One’s ease of use. Excellent (5/5) was found to be the majority rating throughout all judging criteria. The participating surgeons felt that ‘ease of loading’ was the IOLs best quality, with 12 (86%) providing a rating of excellent (5/5). The IOLs worst quality was thought to be ‘ease of placement into the capsular bag’, whereby 6 surgeons provided an excellent (5/5) rating and 1 provided a below average (2/5) rating. Nine (64%) of the surgeons provided an overall rating of excellent (5/5) for the EyeCee® One. The remaining 5 respondents felt it earned an overall rating of good (4/5) (*see* Table 3). One surgeon felt that wound enlargement was necessary during implantation. All of the participating surgeons thought the IOL was clinically acceptable and merited repeat use.

**Table 3:** Surgeons’ observations: 5-point scale (1 being poor, 5 being excellent)

	1	2	3	4	5
Ease of loading	0	0	0	2	12
Ease of introduction	0	0	0	4	10
Control of lens injection	0	0	0	5	9
Ease of placement into capsular bag	0	1	2	5	6
Centration	0	0	1	2	11
Overall rating	0	0	0	5	9

## Discussion

Our observational study evaluated the clinical safety and efficacy of the EyeCee® One. Patient characteristics in our study are reflective of the real-world cataract surgery patient population. This is validated by the similar rates of ocular pathology found in the RCOphth NOD audit 2019 [10]. The EyeCee® One’s ability to improve visual acuity is comparable to other published studies involving preloaded and non-preloaded IOLs. Out of all 152 eyes in our study, 150 (98.7%) had a post-operative monocular CDVA that was equal or better than ‘good’ i.e. ≤ 0.3 logMAR, which is a similar performance to post-operative eyes in recent large multi-center studies involving monofocal IOL implantation [10–12]. The two eyes in our study that failed to achieve a post-operative CDVA ≤0.3 logMAR both had dry AMD with geographic atrophy, which likely impaired their visual acuity outcomes. The mean CDVA of all eyes in our study improved from 0.43 ± 0.43 logMAR pre-operatively, to 0.05 ± 0.11 logMAR post-operatively (*p* < 0.05). Again, this result is comparable to those in recent studies involving similar IOLs [12–15]. A marginally superior post-operative mean CDVA result has been reported in the EUREQUO 2018 annual report for Cataract and Refractive Surgery [16]. However, the smaller percentage of eyes with concomitant ocular pathologies included in this report is a noteworthy factor. Unfortunately, cataract surgery does not guarantee improved visual acuity. One eye (0.66%) that underwent EyeCee® One implantation in our study experienced post-operative visual acuity loss of 0.10 logMAR that was attributed to progression of dry AMD with geographic atrophy. Nevertheless, this is a marginally lower rate than the 0.7% found in the RCOphth NOD audit 2019, whereby loss of visual acuity was associated with concomitant ocular pathology and had no relationship to surgeons’ grade [10]. Our study revealed no concerning safety issues. No serious incidents arose from IOL injection over the trial period (and up to this date of submission). No intraoperative complications were recorded and only two eyes (1.3%) suffered complications 2 weeks post-operatively. These complications (allergic conjunctivitis and entropion) are associated with cataract surgery in general and are unlikely to be related to the choice of IOL or delivery system.

Recent publications have reinforced that preloaded monofocal hydrophobic acrylic IOLs provide an opportunity to make cataract surgery quicker, easier, safer, and therefore more economical. A recent observational study involving 200 routine cataract surgeries showed a significantly decreased mean total case time by 7.7–7.8% when using a preloaded monofocal hydrophobic acrylic IOL rather than a manual IOL delivery process [17]. Preloaded monofocal hydrophobic acrylic IOLs have also performed well in recent studies involving challenging cases of pseudoexfoliation syndrome [18, 19]. The EyeCee® One’s preloaded injector system helps to avoid contamination which may lead to infection and irritation. In addition, its hydrophobic acrylic characteristic ensures gentle and safe unfolding of IOL during procedure, which helps to avoid PCO. Long-term complications were not measured in our study. However, in a study that compared outcomes of EyeCee® One and Acrysof SN60WF implantation, *Leydolt* et al. reported no significant differences in the low rates of decentration after 6 months and the low rates of PCO and Nd:YAG after 3 years [20]. The results from our feedback form have provided insight to operating surgeons’ observations of the EyeCee® One. These observations are consistent with other studies in which authors report similar ease of handling when using hydrophobic acrylic IOLs combined with preloaded delivery systems [21, 22].

Strengths of our study include the use of real-world data and no patients being excluded due to their comorbidities. Together, these factors provide an authentic prevalence of concomitant ocular pathologies and a pre-operative mean CDVA that is more representative of population averages than in other studies [23–25]. Our study was also strengthened by the involvement of multiple surgeons and the excellent success rate of ensuring follow-up. Weaknesses of our study correspond with areas of non-compliance to International Organization for Standardization (ISO) 11,979–7:2018 recommendations for investigations of IOLs [26]. Specifically, the number of subjects, treatment centers, and follow-up intervals restrict our study’s ability to evaluate IOL performance and safety. Additional data that would have added value to the study includes IOL unloading time, incision size after implantation, course of intraocular pressure and presence of postoperative glistenings. Operating surgeons’ observations of other IOLs would also have been valuable as this would have enabled direct comparison to EyeCee® One. Furthermore, a greater number of feedback forms collected would have strengthened our study.

## Conclusions

Our study reports pragmatic data that reveals good to excellent post-operative visual acuity and refractive outcomes in eyes 3 months after EyeCee® One implantation. This is accompanied with very little risk of intraoperative and 2 weeks post-operative complications. The results of this study contribute to the growing evidence of positive outcomes when phacoemulsification is combined with monofocal hydrophobic acrylic IOL implantation via preloaded injector in eyes. More studies are required to further evaluate long-term visual performance and complications.

## Data Availability

The datasets collected and analyzed as part of this study are available from the corresponding author on reasonable request.

## Abbreviations

AMD: Age-related macular degeneration
CDVA: Corrected distance visual acuity
EUREQUO: European Registry of Quality Outcomes
IOL: Intraocular lens
ISO: International Organization for Standardization
logMAR: Logarithm of the minimum angle of resolution
Nd:YAG: Neodymium-doped yttrium aluminium garnet
PCO: Posterior capsule opacification
RCOphth NOD: The Royal College of Ophthalmologists National Ophthalmology Database
SD: Standard deviation
UDVA: Uncorrected distance visual acuity
